# Use of *Plasmodium falciparum* culture-adapted field isolates for *in vitro* exflagellation-blocking assay

**DOI:** 10.1186/s12936-015-0752-x

**Published:** 2015-06-04

**Authors:** Louis-Jérôme Leba, Lise Musset, Stéphane Pelleau, Yannick Estevez, Caroline Birer, Sébastien Briolant, Benoit Witkowski, Didier Ménard, Michael J Delves, Eric Legrand, Christophe Duplais, Jean Popovici

**Affiliations:** Laboratoire de Parasitologie, Institut Pasteur de la Guyane, Cayenne, French Guiana France; Laboratoire ECOFOG (Ecology of Guiana Forests) UMR8172 CNRS-INRA-CIRAD-AgroParisTech-Université des Antilles-Université de la Guyane, French Guiana, France; Direction Interarmées du Service de Santé en Guyane, Cayenne, French Guiana France; Institut de Recherche Biomédicale des Armées, Brétigny sur Orge, France; Malaria Molecular Epidemiology Unit, Institut Pasteur du Cambodge, Phnom Penh, Cambodia; Department of Life Sciences, Imperial College, London, UK

## Abstract

**Background:**

A major requirement for malaria elimination is the development of transmission-blocking interventions. *In vitro* transmission-blocking bioassays currently mostly rely on the use of very few *Plasmodium falciparum* reference laboratory strains isolated decades ago. To fill a piece of the gap between laboratory experimental models and natural systems, the purpose of this work was to determine if culture-adapted field isolates of *P. falciparum* are suitable for *in vitro* transmission-blocking bioassays targeting functional maturity of male gametocytes: exflagellation.

**Methods:**

*Plasmodium falciparum* isolates were adapted to *in vitro* culture before being used for *in vitro* gametocyte production. Maturation was assessed by microscopic observation of gametocyte morphology over time of culture and the functional viability of male gametocytes was assessed by microscopic counting of exflagellating gametocytes. Suitability for *in vitro* exflagellation-blocking bioassays was determined using dihydroartemisinin and methylene blue.

**Results:**

*In vitro* gametocyte production was achieved using two isolates from French Guiana and two isolates from Cambodia. Functional maturity of male gametocytes was assessed by exflagellation observations and all four isolates could be used in exflagellation-blocking bioassays with adequate response to methylene blue and dihydroartemisinin.

**Conclusion:**

This work shows that *in vitro* culture-adapted *P. falciparum* field isolates of different genetic background, from South America and Southeast Asia, can successfully be used for bioassays targeting the male gametocyte to gamete transition, exflagellation.

**Electronic supplementary material:**

The online version of this article (doi:10.1186/s12936-015-0752-x) contains supplementary material, which is available to authorized users.

## Background

Among the actions required for malaria elimination, blocking the transmission of *Plasmodium* parasites from human to mosquitoes is critical [1]. Passage through the vector is an obligatory step for the parasite to continue its life cycle and it relies exclusively on the most mature forms of the sexual stages, stage V gametocytes. Mosquito feeding assays remain the gold standard to evaluate transmission-blocking strategies but require resource-intensive techniques. To circumvent these technical difficulties, several *in vitro* transmission-blocking bioassays targeting the sexual stages of the parasite have been described with different endpoints and various interpretative values [2, 3]. Although the outputs of these *in vitro* assays are difficult to transpose *in vivo*, the most clinically relevant assay should be able to evaluate the ability of a compound to either kill stage V gametocytes (viability assay) or inhibit their ability to differentiate into later mosquito stages (functional maturity assay). It is possible to induce *in vitro* gamete formation from *Plasmodium falciparum* male and female gametocytes where male gametocytes undergo major differentiation leading to the production of eight mobile gametes, by a process called exflagellation, which is easily observed visually.

Currently, the majority of *P. falciparum in vitro* transmission-blocking studies use a handful of reference laboratory strains, isolated decades ago. While these strains are useful to normalize high throughput screenings, results should be verified on natural parasites that have been selected after years of multiple drug exposures and hence are likely to display differential drug responses compared to reference strains. In addition, it is well known that gametocyte production capacity is lost over time of *in vitro* culture [2, 4]. Laboratories must therefore rely on precious stocks of cryopreserved isolates with minimal passage since in culture. Adaptation of *P. falciparum* isolates from patients to *in vitro* blood-stage culture is routinely performed. Studies describing the use of field isolates for *in vitro* gametocyte cultures have been published in the early 1980s and have led to the selection of the current reference strains [4–8]. Nowadays using circulating parasites after culture adaptation for transmission-blocking assay is rarely performed. Some studies have shown that they can be used in gametocyte viability assay [9] or for experimental infections of mosquitoes [10], however their use for exflagellation-blocking bioassays, reporting *in vitro* functional maturity of gametocytes has not been reported yet. A requirement for their use in such assay is to be able to produce functional gametocytes in high enough number to be meaningful in a bioassay.

The objective of the work presented here was therefore to determine if culture-adapted field isolates could be used for recently developed *in vitro* exflagellation-blocking bioassays [11].

## Methods

### Reference strain and field isolates

The *P. falciparum* South American chloroquine-resistant strain 7G8 (MRA-926) has been obtained from the MR4. *Plasmodium falciparum* isolates were collected from mono-infected patients seeking treatment in 2013 in French Guiana (Q206 and Q188) and in 2014 in Cambodia (6831 and 6836).

### *In vitro* culture adaptation

Culture adaptation of isolates was performed using standard protocols [12, 13]. Briefly, after removal of plasma, the red blood cell (RBC) pellet was washed three times in RPMI 1640 supplemented with gentamicin (Gibco-Life Technologies SAS, France) and placed in culture medium (RPMI 1640, 0.5 % AlbuMAX II (Gibco-Life Technologies SAS, France), 2 % decomplemented human plasma) at 4 % haematocrit at 37 °C in 5 or 10 % O_2_, 5 % CO_2_, rest of N_2_ atmosphere. Parasitaemia was checked daily and kept below 2 % by dilutions with fresh RBC and medium. Field isolates were considered adapted to *in vitro* conditions after three weeks of uninterrupted culture. After culture adaptation, asexual blood-stage sensitivity to anti-malarials was determined using the [^3^H]-hypoxanthine incorporation assay [14].

### *In vitro* gametocyte production and maturation

Gametocyte cultures were performed following published protocols [2, 11]. Briefly, asexual cultures with a parasitaemia of ~5 % were used to seed gametocyte cultures at 0.5–1 % parasitaemia and 4 % haematocrit under 5 or 10 % O_2_, 5 % CO_2_, rest of N_2_ atmosphere. Culture medium (RPMI medium with 25 mM HEPES, 50 mg/L hypoxanthine, 2 g/L sodium bicarbonate, 10 % human serum) was replaced daily but without any further addition of RBCs and critically, temperature was maintained at all time at 37 °C.

Gametocytogenesis was evaluated morphologically using Giemsa-stained blood films. Stage V male gametocyte functional maturity was assessed by observation of exflagellation in wet preparation under bright-field microscope. A 30-μL aliquot of gametocyte culture was briefly centrifuged, the cell pellet was resuspended in 15 μL of ookinete medium (RPMI medium with 25 mM HEPES, 50 mg/L hypoxanthine, 2 g/L sodium bicarbonate, 100 μM xanthurenic acid, 10 % human serum) and then introduced into a chamber of a FastRead disposable haemocytometer slide (Immune Systems). Exflagellation centres were observed at 10 × or 20 × magnification.

### Exflagellation-blocking bioassay

Exflagellation-blocking assays were performed according to published protocols [11]. Assays were performed using gametocyte cultures providing high enough exflagellation centres for meaningful measures (>30 in five 10 × microscopy fields).

To show the suitability of field isolates for exflagellation-blocking assays, the activity of 1 μM of dihydroartemisinin (DHA), methylene blue (MeBlue) and chloroquine (CQ) was evaluated. Assays were carried out in 1.5-mL tubes containing 170 μL of gametocyte culture medium with 1 μM of drugs dissolved in either DMSO or methanol. Thirty μL of stage V mature gametocyte was dispensed into each assay tube. Tubes were then placed into a 37 °C incubator. After 24 h, exflagellation was induced by temperature drop to 25 °C and replacing culture medium with ookinete medium. The number of exflagellation centres were recorded and compared to controls (DMSO or methanol). Activity was expressed as percentage of exflagellation inhibition compared to controls.

## Results

**Fig. 1:**
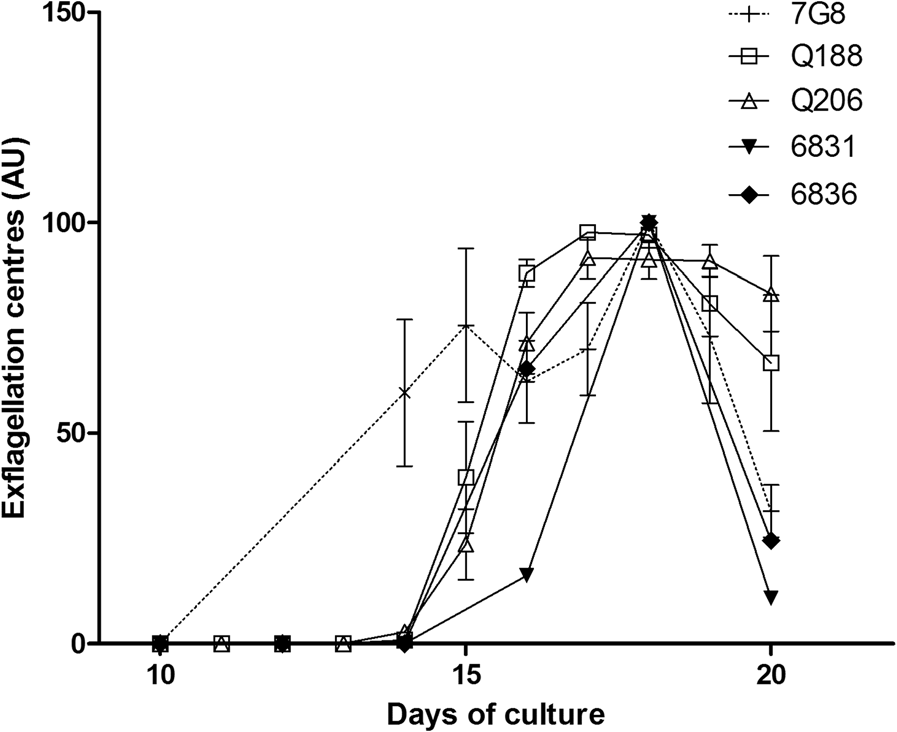
Exflagellation levels over days of gametocyte culture (arbitrary unit). For each independent culture, exflagellation centres values are normalized to the highest value of the culture (data are presented in Additional file 1). The mean and SEM of three cultures is shown for 7G8 and the two French Guiana strains (Q188 and Q206) while a single culture for each Cambodian isolate is represented (6831 and 6836)

The protocols were first optimized by using the South American isolates prior to being verified with the Southeast Asian isolates. Gametocyte development was straightforward at the first attempt. Initially however, although stage V gametocytes were clearly identified in the cultures, no exflagellation was observed. This suggests that bioassays with a read-out based only on morphological development of gametocytes do not report on their onward functional viability, therefore they may be underpowered. As already observed by others [2, 4], the age of uninfected RBC when initiating the gametocyte culture was a critical factor for successful maturation: RBC stored at 4 °C more than ~ two to 3 days rapidly compromise maturation. Additionally, variability in human serum was found also to be a critical point for gametocyte maturation, so pools from different donors of serum were used when possible. Using fresh uninfected RBC, fully mature gametocytes capable of exflagellation were generated for all isolates. For all isolates and 7G8, gametocyte development time was similar and maturity peaked at ~16–18 days of culture, slightly longer than what is usually reported for 3D7, ~12–14 days (Fig. 1) [2, 11].

It is important to note that all the experiments using 7G8 and the South American isolates were performed in a different laboratory (Institut Pasteur de la Guyane, Cayenne) than those using the Cambodian isolates (Institut Pasteur du Cambodge, Phnom Penh) showing good reproducibility in the protocols that can be easily implemented in laboratories doing *P. falciparum in vitro* culture adaptation.

**Fig. 2:**
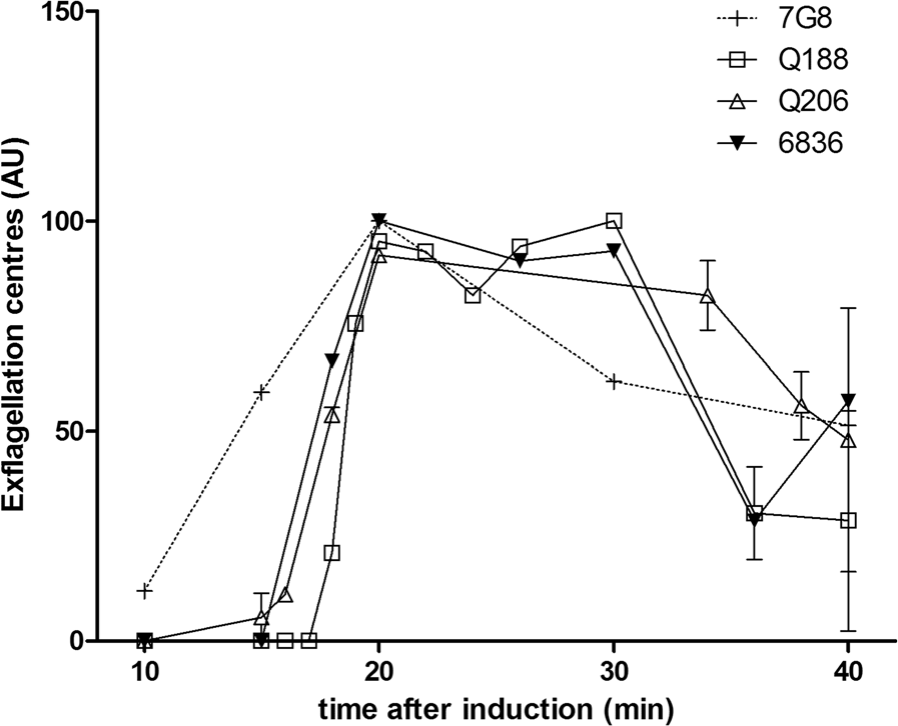
Exflagellation levels over time after temperature drop and addition of xanthurenic acid of gametocyte cultures (arbitrary unit). For each independent culture, exflagellation centres values are normalized to the highest value of the culture (data are presented in Additional file 1). The mean and SEM of two independent culture is shown for the two French Guiana strains (Q188 and Q206) while a single culture for the Cambodian isolate (6836) and the reference strain 7G8 are represented

Exflagellation is a time-dependent process [15]. Within 20 min after induction, for all field isolates and the reference strain 7G8, a plateau is reached allowing consistent measurements for ~10 min (Fig. 2). This is similar to what has previously been observed with 3D7 [11].

Although the patterns of gametocyte development and time for exflagellation are similar for all isolates and laboratory strains, there are variations in the amount of exflagellation centres from one isolate to another (see Additional file 1) as already observed by others [4]. For example, in French Guiana, the isolate Q206 consistently gave higher number of exflagellation than Q188. Nevertheless, for all the isolates, using gametocyte cultures at maturity, the numbers of exflagellation centres obtained were high enough to allow significant measures in exflagellation-blocking bioassay (from ~35 to >100, see Additional file 1).

Based on those observations, a previously described exflagellation-blocking protocol was adapted to the four field isolates and 7G8 [11]. Exflagellation centres were recorded between 20 and 30 min after induction of 18 days-old gametocyte cultures. As a proof-of-concept, the activity of DHA and MeBlue was evaluated as both compounds have been previously reported to block exflagellation of 3D7 [11]. As negative control, CQ was used on a sub-set of the strains [16]. Mature gametocytes were incubated 24 h in presence of 1 μM of each drug before exflagellation was induced. All isolates responded similarly to the compounds with 1 μM DHA giving 78–99 % inhibition and consistently near total inhibition with 1 μM of MeBlue (Table 1). As expected, CQ had no effect on exflagellation in any isolate tested.

**Table 1:** Exflagellation-blocking activity^a^ of dihydroartemisinin (DHA), methylene blue (MeBlue) and chloroquine (CQ) against *Plasmodium falciparum* field isolates and the 7G8 reference strain

	7G8	Q188	Q206	6831	6836
DHA	99 ± 1.3	90 ± 8.3	78 ± 7.2	84 ± 12.2	79 ± 11.6
MeBlue	100	100	99 ± 1.7	100	99 ± 2.1
CQ	−1 ± 10.8	ND	5.7 ± 10.2	ND	7.3 ± 19.2

^a^Expressed as a percentage of inhibition compared to the drug-free control (mean ± SEM, *n* = 3)

## Conclusion

This work reports that freshly culture-adapted field isolates, from various geographical origins and genetic background can produce functionally mature gametocytes *in vitro* in high enough numbers to be successfully used in exflagellation-blocking bioassays. Moreover, the assay was sensitive enough to observe inter-strain differences, which might be of significant interest for comprehension of malaria transmission in the field. Indeed, the Q188 isolate is chloroquine-sensitive (IC_50_ = 34.93 nM ± 1.91) and consistently produced fewer gametocytes *in vitro* than the Q206 isolate that is chloroquine-resistant (IC_50_ = 483.36 nM ± 61.25). Of course, given the low number of isolates, no conclusion can be drawn based on this work and further studies are required to evaluate a possible link between chemosensitivity and *in vitro* gametocytogenesis. In addition, at least one cryopreservation cycle did not impair the capacity of the field isolates in producing mature gametocytes. However, for how long in culture this capacity will be maintained before being lost, as expected remains to be evaluated. Finally, the infectivity to mosquitoes of the mature gametocytes should be assessed to transpose the *in vitro* results to an *in vivo* model of transmission. Capacity building for experimental infections of mosquitoes is currently being undergone in Institut Pasteur, both in French Guiana and in Cambodia, and will allow such experiments in a near future.

## Additional file

Additional file 1:
**Absolute and normalized exflagellation values.** Dataset including exflagellation values over days of culture and exflagellation values over time after induction.
